# Red Oranges and Olive Leaf Waste-Derived Bioactive Extracts Promote Adipocyte Functionality In Vitro

**DOI:** 10.3390/nu16121959

**Published:** 2024-06-19

**Authors:** Maria Gulisano, Valeria Consoli, Valeria Sorrenti, Luca Vanella

**Affiliations:** 1 Department of Drug and Health Sciences, University of Catania, 95125 Catania, Italy; maria.gulisano@hotmail.it (M.G.); valeria_consoli@yahoo.it (V.C.); lvanella@unict.it (L.V.); 2 CERNUT—Research Centre for Nutraceuticals and Health Products, University of Catania, 95125 Catania, Italy

**Keywords:** adipocytes, 3T3-L1, inflammation, palmitic acid, erastin, health, nutraceuticals

## Abstract

Obesity is increasingly prevalent worldwide and is linked to metabolic diseases, such as insulin resistance (IR) and type 2 diabetes mellitus (T2DM), due to excessive free fatty acids (FFAs). Although lifestyle changes are effective, they often prove to be insufficient as initial treatments for obesity. Additionally, while surgical and pharmacological interventions are available, they are not entirely safe or effective. Recently, interest has grown in utilizing food waste and plant-derived phenolic compounds for their health benefits, presenting a promising avenue for managing obesity and its related disorders. Indeed, many studies have examined the potential inhibitory effects of the natural extract on adipocyte differentiation and lipid accumulation. This study focused on the evaluation of the effects of standardized extracts obtained from red oranges and olive leaf waste on 3T3-L1 murine pre-adipocyte and adipocyte functionality. Red orange extract (ROE) and olive leaf extract (OLE), alone and in combination, were tested to assess their anti-obesity and anti-inflammatory effects, as well as their potential therapeutic benefits. Three in vitro models were established to investigate the effects of the extracts on (I) adipocyte differentiation; (II) mature and hypertrophic adipocytes challenged with palmitic acid (PA) and erastin (ER), respectively; and (III) erastin-induced cytotoxicity on pre-adipocytes.

## 1. Introduction

Obesity is recognized as a global epidemic and a significant risk factor for several health conditions, including insulin resistance, type 2 diabetes mellitus (T2DM), cardiovascular diseases (CVDs), immune dysfunction, non-alcoholic fatty liver disease (NAFLD), various types of cancer, and dyslipidemic conditions [1,2]. The accumulation of white adipose tissue (WAT), associated with the storage of surplus energy as triacylglycerols in adipocytes, is a defining characteristic of obesity related to both adipocyte hypertrophy (increase in size of existing adipocytes) and hyperplasia (pre-adipocyte differentiation into mature adipocyte by de novo adipogenesis) [3,4]. WAT serves as an energy reservoir and, in addiction, functions as the major endocrine organ, secreting adipokines and cytokines into the bloodstream. Adipokines play roles in several metabolic and physiological signaling pathways, regulation of insulin signaling, glucose uptake, oxidation of fatty acids, and more energy-related processes. Released cytokines can modulate the inflammatory response, depending on their intrinsic activity as pro- or anti-inflammatory [1,2]. Pre-adipocytes, the primary cellular constituents of WAT, undergo adipogenesis to mature and become functional adipocytes following both morphology and gene expression changes [5].

Adipogenesis refers to the process by which precursor cells proliferate and differentiate into mature adipocytes. This intricate process is tightly regulated by various signaling pathways and transcriptional cascades. The final stage of differentiation is correlated with changes in the morphological and biochemical features of mature adipocytes, differing from fibroblast-like pre-adipocytes. Consequently, the identification of compounds with anti-adipogenic properties that can control adipocyte differentiation represents a potential innovative therapeutic strategy for metabolic disorders. The development of an inflammatory state, which is thought to be the underlying cause of insulin resistance in obesity, is sustained by the malfunctioning of adipokine release and the accumulation of free fatty acids. It is important to note that these changes are associated with the location of fat deposition, with visceral fat accumulation being primarily responsible for dysfunctional adipose tissue, metabolic issues, and elevated cardiovascular risk, even in individuals with normal weight [6]. Individuals with obesity and T2DM typically have elevated levels of non-esterified free fatty acids (FFAs) in their blood, particularly palmitic acid, which is one of the saturated fatty acids frequently found in circulation. Palmitic acid (PA) is known to impair insulin signaling by inhibiting its action in hepatocytes, myocytes, and adipocytes. Additionally, exposure to palmitic acid has been linked to pancreatic beta cell dysfunction, contributing to lipotoxicity [7].

Despite the availability of various surgical and pharmacotherapeutic interventions, there are currently no completely safe and effective treatments for weight management. Lifestyle changes, including diet modifications and increased physical activity, are considered the most viable options [8]. Adopting natural extracts may offer a promising strategy for managing obesity and its associated disorders. Various studies have explored the potential inhibitory effects of natural extracts on adipocyte differentiation and lipid accumulation. Indeed, plant-based medications are often regarded as the primary approach to maintaining health and preventing diseases and their complications [4,9,10].

In recent times, there has been a surge of interest in exploiting food waste, particularly fruit- and vegetable-derived byproducts, to extract bioactive compounds for developing new formulations that have the potential to exhibit considerable positive effects on public health [11].

The current study focused on red oranges and olive leaf waste as raw materials to produce standardized extracts obtained through an industrial extraction process and the recovery of bioactive molecules using adsorbent resins from byproducts; the obtained extracts, abundant in bioactive compounds such as polyphenols, were previously tested, and the results demonstrated their ability to reduce FFA accumulation and act as cholesterol-lowering agents [12].

Impaired iron metabolism can result in abnormal differentiation of adipose tissue, which can lead to metabolic disorders [13]. Indeed, high iron levels may contribute to the continuous release of adipokines and promote an inflammatory environment, redirecting immune responses and promoting obesity occurrence [14]. Obesity-related persistent inflammation is characterized by the disproportionate release of FFAs and proinflammatory cytokines from adipocytes; this has led to the hypothesis of a connection between obesity and ferroptosis [15]. Ferroptosis, which was identified in 2012, is an iron-dependent cell death mechanism that is distinct from apoptosis, necrosis, and other cellular death mechanisms. Although ferroptosis has been involved in several disease development mechanisms, adipose tissue research still lacks related, well-defined data. Since redox homeostasis is crucial for pre-adipocyte recruitment, a preliminary attempt to investigate the pro-oxidant effect of the well-known ferroptosis inducer erastin was made [16].

The aim of this study was to elucidate the anti-obesity and anti-inflammatory effects and the therapeutic potential of ROE and OLE, alone and in combination, in 3T3-L1 cells.

For this purpose, three in vitro models were established to examine the effects of extracts on (I) adipocyte differentiation; (II) mature and hypertrophic adipocytes challenged with PA and ER, respectively; and (III) erastin-induced cytotoxicity on pre-adipocytes.

## 2. Materials and Methods

The powered red orange extract (ROE) (batch no. 6/21) and the powered olive leaf extract (OLE) (batch no. E21/2691) utilized in this work were prepared by Medinutrex (Catania, Italy). The ROE and OLE were used for the analysis as previously published [12]. The impact of various concentrations (ROE: 0.5–1–3 mg/mL; OLE: 0.5–1–3 mg/mL; COMBO 1: ROE 0.5 + OLE 0.5 mg/mL; COMBO 2: ROE 1 + OLE 1 mg/mL; and COMBO 3: ROE 3 + OLE 3 mg/mL) was assessed on cell viability, and ROE and OLE 1 mg/mL were chosen as the optimal and non-toxic concentrations for the subsequent experiments.

### 2.1. Assessment of Antioxidant Activity in a Cell-Free System

#### FRAP

The potential of the antioxidant activity of the ROE and OLE was determined by employing the ferric reducing antioxidant power assay (FRAP) in a cell-free system, an in vitro model where biochemical reactions occur independently of living cells. The analysis was conducted following the procedure described in the FRAP assay kit (MAK369, Sigma-Aldrich, St. Louis, MO, USA). In this method, antioxidants were used as reductants in a redox-linked colorimetric reaction, wherein Fe^3+^ is reduced to Fe^2+^. The absorbance was measured spectrophotometrically at 590 nm, and color intensity results were proportional to FRAP in the sample. Three replicate wells were used for each group. The results are expressed as µM ferrous equivalents and calculated as follows:

(B × D/V)/1000,

where B = ferrous ammonium sulphate amount from the standard curve (nmol); D = sample dilution factor; and V = sample volume added into the reaction well (μL).

### 2.2. Cell Culture Experiments

#### 2.2.1. Cell Culture and Differentiation of 3T3-L1 Pre-Adipocytes

The murine pre-adipocyte cell line 3T3-L1 was obtained from the American Type Culture Collection (ATCC; Rockville, MD, USA). Once reached confluence, to induce differentiation, The medium was replaced with an adipogenic one, as already reported by Ballistreri et al. [17]. The cultures were maintained at 37 °C and 5% CO_2_ in an incubator, and the medium was changed every 3 days in the presence or absence of the ROE and OLE, alone and in combination.

#### 2.2.2. Cell Viability Assay

3T3-L1 cells were seeded in a 96-well cell culture plate and were treated for 48 h with different concentrations of the extracts alone or in combination (ROE: 0.5–1–3 mg/mL; OLE: 0.5–1–3 mg/mL; COMBO 1: ROE 0.5 + OLE 0.5 mg/mL; COMBO 2: ROE 1 + OLE 1 mg/mL; and COMBO 3: ROE 3 + OLE 3 mg/mL).

Afterward, to establish an in vitro model of ferroptosis, 3T3-L1 pre-adipocytes were cultured in a 96-well cell culture plate, pre-treated with the ROE and OLE, alone and in combination, and incubated for 24 h. Then, the cells were challenged with erastin (1 μM) in the presence or absence of the extracts for 48 h.

Cell viability was assessed by the MTT assay, which was carried out following the previously described procedure by Consoli et al. [18]. Eight replicate wells were utilized for each group.

#### 2.2.3. Oil Red O Staining

3T3-L1 pre-adipocytes were plated in a 24-well plate. After 24 h, the medium was replaced with adipogenic medium, and the cells were differentiated for 13 days with ROE–OLE alone or in combination (ROE: 0.5–1 mg/mL; OLE: 0.5–1 mg/mL; COMBO 1: ROE 0.5 + OLE 0.5 mg/mL; and COMBO 2: ROE 1 + OLE 1 mg/mL).

For a second analysis, 3T3-L1 cells were seeded in a 24-well cell culture plate and maintained for 5 days with adipogenic medium. Then, the cells were pre-treated with the ROE and OLE, alone and in combination, and incubated for 24 h. Finally, the differentiated adipocytes were treated with PA (0.5 mM) or ER (1, 5, 10 μM) in the presence or absence of the extracts for 48 h.

To calculate the quantity of lipid droplets, at the end of differentiation, in both experimental conditions, Oil Red O staining was performed as previously reported by Ballistreri et al. [17]. The analysis was carried out in triplicate.

#### 2.2.4. Palmitic Acid–Bovine Serum Albumin Complex Preparation

Palmitic acid (Sigma-Aldrich, St. Louis, MO, USA) was dissolved in NaOH 0.1 M. BSA (Beyotime Biotechnology, Shanghai, China) was dissolved in DMEM to form a 12% BSA solution. For palmitic acid in conjunction with BSA, 12% BSA was added to the palmitic solution and conjugated by ultrasonic treatment. Following an incubation period of 1 h at 50 °C with continuous shaking, the PA–BSA complexes should be clear and were cooled at room temperature, filtrated through a membrane filter (0.22 μm), and stored at +4 °C for further use.

#### 2.2.5. Sircol Collagen Assay

The Sircol collagen assay (Biocolor, Belfast, UK) was utilized to measure total soluble collagen in cell culture supernatants, as previously described by Raffaele et al. [19], with some adjustments. For these experiments, confluent cells in 24-well plates were incubated for 13 days under the same abovementioned conditions for Oil Red O Staining, and the analysis was conducted in triplicate.

#### 2.2.6. RNA Extraction and qRT-PCR Analysis

Total RNA was isolated from 3T3-L1 differentiated adipocyte cells using TRIzol reagent (Invitrogen, Carlsbad, CA, USA). It was subsequently converted into cDNA through an Applied Biosystem (Foster City, CA, USA) reverse transcription reagent.

Quantitative Real-time PCR analysis was conducted, as formerly described by Vanella et al. [20], to evaluate the gene expression of fatty acid synthase (FAS), diacylglycerol acyltransferases 1 and 2 (DGAT-1 and DGAT-2, respectively), sterol regulatory element-binding protein-1c (SREBP-1C), collagen type I alpha 1 (COL1A1), interleukin-6 (IL-6), fatty acid transport proteins 1 and 4 (FATP-1 and FATP-4, respectively), and heme oxygenase-1 (HO-1). The analysis was executed in triplicate.

### 2.3. Statistical Analysis

Each analysis constituted the results of a minimum of three distinct experiments. Fisher’s method was utilized to determine the statistical significance (*p* < 0.05) of the differences between the experimental groups.

For a comparison of treatment groups, the null hypothesis was tested by either a single-factor analysis of variance (ANOVA) for multiple groups or an unpaired *t*-test for two groups, and the data were presented as the means ± SEMs.

## 3. Results

### 3.1. Iron-Reducing Activities of the ROE and OLE and Their Effect on Cell Viability

**Figure 1:**
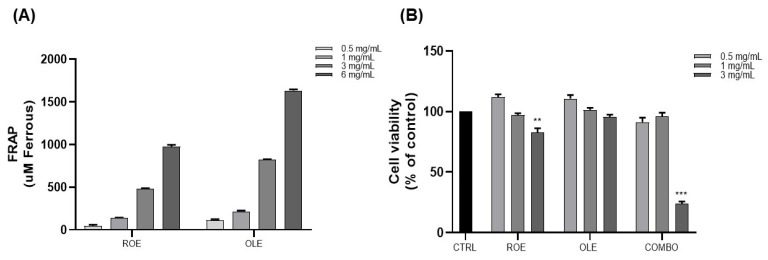
(**A**) Evaluation of ROE and OLE’s antioxidant activity at different concentrations by the FRAP assay. The results are expressed as μM ferrous equivalent. (**B**) The effects of ROE and OLE, alone and in combination, on the cell viability of 3T3-L1 cells. Each value represents the mean ± SEM (** *p* < 0.005; *** *p* < 0.0005 vs. CTRL). The data are indicated as means ± SEMs.

ROE and OLE antioxidant activity were investigated in a cell-free model by the FRAP assay (Figure 1A). The ROE and OLE were examined at different concentrations (0.5–1–3–6 mg/mL), and both showed a strong dose-dependent antioxidant effect, confirming previously observed scavenger activity by the DPPH assay [12].

To determine the optimal concentration of extracts, cytotoxicity was assessed for the ROE and OLE, alone and in combination, in 3T3-L1 pre-adipocytes cells by the MTT assay. Cells were treated with different concentrations of extracts (ROE: 0.5–1–3 mg/mL; OLE: 0.5–1–3 mg/mL; and combination treatment 1:1 as follows: COMBO 1: 0.5 mg/mL; COMBO 2: 1 mg/mL; and COMBO 3: 3 mg/mL). As displayed in Figure 1B, ROE and OLE concentrations of 0.5–1 mg/mL, alone and in combination, did not affect the viability of 3T3-L1 cells. However, the highest analyzed concentration (3 mg/mL) exhibited an in vitro cytotoxic effect after 48 h of treatment. Therefore, concentrations of 0.5–1 mg/mL of the ROE and OLE were used for subsequent studies.

### 3.2. The Effects of the ROE and OLE on Lipid and Collagen Accumulation during Adipogenesis

**Figure 2:**
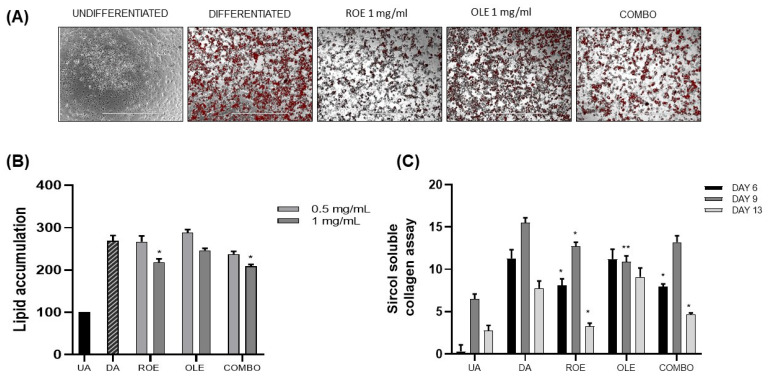
(**A**) Representative images of lipid accumulation stained with Oil Red O Staining in 3T3-L1 cells. (**B**) The effect of the ROE and OLE, alone and in combination, (0.5–1 mg/mL) on lipid accumulation in 3T3-L1 cells during 13 days of differentiation (* *p* < 0.05 vs. DA). (**C**) Evaluation of the ROE and OLE on total soluble collagen by the Sircol collagen assay at different time points: 6, 9, and 13 days (* *p* < 0.05, ** *p* < 0.005 vs. DAs). The data are indicated as means ± SEMs. (**A**) Scale bar 1000 μm is reported in each panel. All images are original and unedited.

To examine the effect of ROE and OLE, alone and in combination, during adipocyte differentiation, 3T3-L1 pre-adipocytes were maintained in adipogenic medium containing extracts at different concentrations (ROE: 0.5–1 mg/mL; OLE: 0.5–1 mg/mL; COMBO 1: 0.5 ROE + 0.5 OLE mg/mL; and COMBO 2: 1 ROE + 1 OLE mg/mL) for 13 days. On day 13, Oil Red O staining (Figure 2A) was carried out to assess the accumulation of lipid droplets. Figure 2B shows that ROE and OLE treatment at concentrations of 0.5 mg/mL did not display any significant reduction in lipid accumulation compared to differentiated adipocytes (DAs). However, ROE and OLE treatment at concentrations of 1 mg/mL was capable of slightly reducing lipid droplets in 3T3-L1 cells compared to DAs. Concerning the COMBO treatments, the highest combination showed a synergistic effect on reducing lipid droplet accumulation. Therefore, ROE and OLE, alone and in combination, were used at concentrations of 1 mg/mL for subsequent experiments.

Total soluble collagen was quantified in 3T3-L1 cell supernatants using the Sircol collagen assay. Supernatants from the cell cultures were collected at various time points: 6, 9, and 13 days of adipocyte differentiation (Figure 2C). The DAs showed a higher accumulation of collagen at 9 days of differentiation, compared to 6 days, followed by a significant reduction at 13 days. ROE and COMBO showed a reduction of collagen during each time point compared to OLE treatment.

### 3.3. The Effects of the ROE and OLE on Palmitic Acid-Derived Inflammation

**Figure 3:**
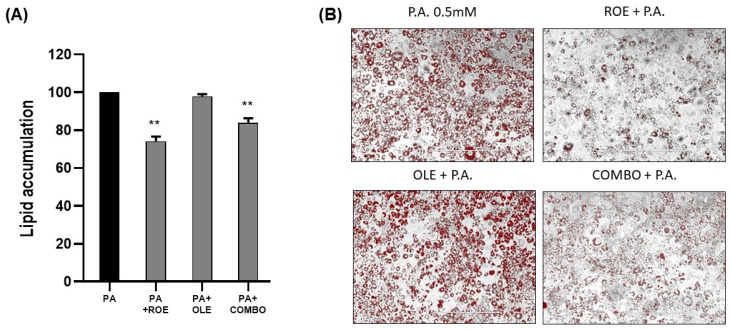
(**A**) The effects of the ROE and OLE, alone and in combination, on lipid droplet accumulation following exposure to PA 0.5 mM (** *p* < 0.005 vs. PA). (**B**) Representative images of Oil Red O’ staining on 3T3 cells co-treated for 48 h with PA 0.5 mM and ROE and OLE, alone and in combination. The data are indicated as means ± SEMs. (**B**) Scale bar 400 μm is reported in each panel. All images are original and unedited.

To establish an in vitro model of inflammation, PA (0.5 mM) was added for 48 h to differentiated 3T3-L1 cells, which were pre-treated with ROE–OLE, alone and in combination, for 24 h. As displayed in Figure 3A,B, ROE and COMBO were able to significantly reduce lipid droplet accumulation induced by treatment of differentiated adipocytes with a non-cytotoxic concentration of PA. The PA treatment was not able to significantly increase lipid accumulation compared to the untreated control. On the other hand, no change in lipid accumulation following OLE treatment was observed compared to PA-treated cells.

**Figure 4:**
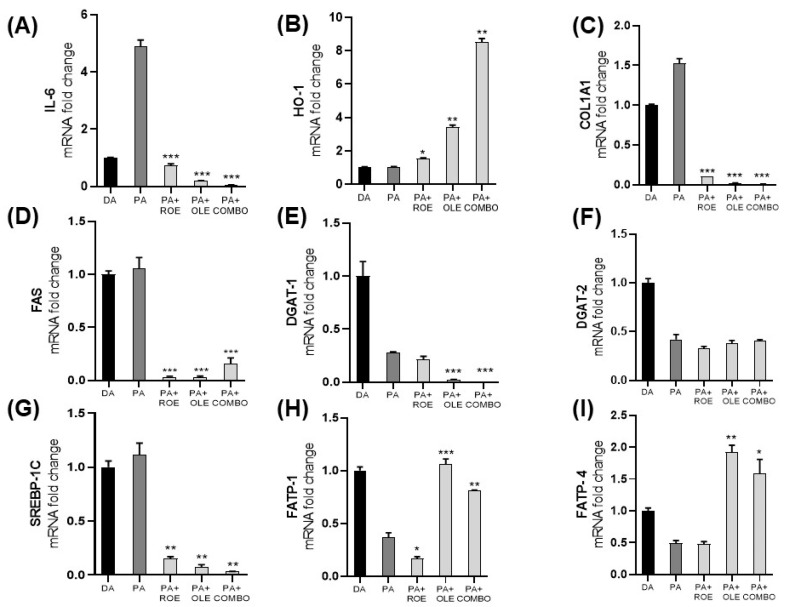
Gene expression measured by qRT-PCR in differentiated adipocytes following inflammation model establishment with PA and co-administration with the ROE and OLE, alone and in combination. mRNA levels of IL-6 (**A**), HO-1 (**B**), COL1A1 (**C**), FAS (**D**), DGAT-1 (**E**), DGAT-2 (**F**), SREBP-1C (**G**), FATP-1 (**H**), FATP-4 (**I**) were evaluated. (* *p* < 0.05; ** *p* < 0.005; *** *p* < 0.0005 vs. PA). The data are indicated as means ± SEMs.

For the purpose of investigating the anti-inflammatory effects of the extracts, gene levels related to proinflammatory cytokine interleukin 6 (IL-6), the antioxidant enzyme heme-oxygenase-1 (HO-1), collagen type I alpha 1 (COL1A1), and lipid metabolism such as fatty acid synthase (FAS), diacylglycerol acyltransferases 1 and 2 (DGAT-1 and DGAT-2, respectively), sterol regulatory element-binding protein-1c (SREBP-1C), and fatty acid transport proteins 1 and 4 (FATP-1 and FATP-4, respectively) were measured by RT-PCR in differentiated adipocytes exposed to PA (Figure 4). Co-treatment of PA and the extracts showed a significant reduction of IL-6 (panel A), which represents one of the main proinflammatory markers. Additionally, the cytoprotective HO-1 enzyme levels (panel B) were evaluated, showing the ROE and OLE synergistic ability to increase HO-1 following exposure to PA. The effect of ROE and OLE obtained on the Sircol assay were confirmed by a significant decrease in the COL1A1 gene (panel C) in differentiated adipocytes exposed to PA. As shown in Figure 4D,E,G, both extracts and their combination significantly decreased FAS, DGAT-1 and SREBP-1C levels, confirming the anti-obesity effects of ROE and OLE; additionally, the latter, alone and in combination, increased FATP-1 and FATP-4 levels (Figure 4H,I), suggesting the activation of fatty acid beta-oxidative process. Although DGAT-2 and DGAT-1 levels were reduced by PA, no change was observed following ROE and OLE treatment for isoform 2 (Figure 4F).

### 3.4. Evaluation of ROE and OLE Effects on Erastin-Challanged Pre-Adipocytes and Mature Adipocytes

**Figure 5:**
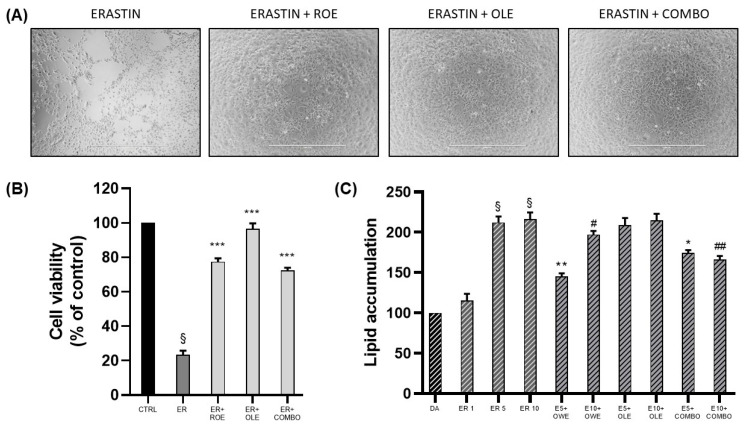
(**A**) Representative images of cell viability on pre-adipocytes co-treated for 48 h with ER 1 μM, ROE, and OLE, alone and in combination. (**B**) The effect of ROE and OLE, alone and in combination, on cell viability after exposition to ER 1 μM (§ *p* < 0.0005 vs. CTRL; *** *p* < 0.0005 vs. ER). (**C**) The effect of ROE and OLE, alone and in combination, on lipid accumulation on differentiated adipocytes challenged with erastin (1–5–10 μM) (§ *p* < 0.0005 vs. DA; * *p* < 0.05; ** *p* < 0.005 vs. ER 5; # *p* < 0.05; ## *p* < 0.005 vs. ER 10). The data are indicated as means ± SEMs. (**A**) Scale bar 1000 μm is reported in each panel. All images are original and unedited.

Firstly, a model of oxidative stress-induced cell death on 3T3-L1 pre-adipocytes was established. The cells were pre-treated for 24 h with ROE–OLE, alone and in combination, and subsequently exposed to erastin (1 μM) for 48 h. Figure 5A,B show that erastin, compared to the control, significantly reduced cell viability. On the other hand, a remarkable recovery was observed in cell vitality following the co-treatment of erastin with ROE and OLE, alone and in combination, compared to erastin.

Interestingly, the preliminary results obtained following erastin treatment on differentiated adipocytes showed no cytotoxic effect compared to pre-adipocytes. However, we decided to investigate the potential effect of erastin on lipid accumulation. As displayed in Figure 5C, erastin at its lowest concentration did not produce any increase in oil red quantification, while both erastin concentrations of 5 and 10 μM were able to significantly increase adipocyte lipid accumulation; thus, combination treatments were performed only with effective ER concentrations. The co-administration of ER and COMBO resulted in a significant reduction in lipid droplet content; however, the results obtained for single extract administration highlighted a major contribution of ROE as it showed lower lipid content quantification compared to OLE.

## 4. Discussion

The commitment and differentiation of pre-adipocytes involve intricate processes elucidated through various in vitro models that facilitate a deeper comprehension of adipogenesis and adipocyte dysfunction linked with obesity and T2DM [21].

In addition to changes occurring within the pancreatic islets, chronic inflammation and obesity stand out as primary causes of insulin resistance, leading to dysfunction in adipose tissue. Adipose tissue plays a crucial role in the immune response by releasing immune system modulators like cytokines and blood clotting factors.

Chronic inflammation and dysregulation of cytokine signaling can lead to irregularities in the deposition of fat and energy metabolism [22]. Sustained positive energy balance prompts the enlargement of present adipocytes (hypertrophy) and/or a rise in the number of pre-adipocytes and adipocytes (hyperplasia) to accommodate surplus energy consumption [23]. When these alterations become significantly pronounced and unmanageable, dysfunction may result in insulin resistance not only within adipose tissue but also in the liver, muscles, and pancreas [22]. Dysfunctional adipocytes produce and release cytokines, including IL-6, tumor necrosis factor α (TNFα), and monocyte chemoattractant protein 1 (MCP-1), which trigger inflammation within adipose tissue, thereby promoting cellular aging, a process also known as cell senescence.

The accumulation of new triacylglycerol (TAG; lipogenesis), the breakdown of formerly stored TAG (lipolysis), and the recruitment of new functional adipocytes (adipogenesis) collectively influence body fat, maintaining an overall balance [23].

Pharmacological and surgical interventions offer effective weight reduction but are often accompanied by side effects. Consequently, there is a demand for identifying safe and efficient alternative treatments or prevention protocols. In recent years, attention has turned to food waste, plants, and bioactive phytochemicals as encouraging sources for identifying novel pharmacological agents to address obesity and its associated complications [24]. Plant-derived natural compounds, including phenolic compounds, are gaining attraction as therapeutic agents. They have shown the capacity to influence the physiological and molecular pathways that regulate adiposity, probably preventing obesity and its associated metabolic disorders [25]. However, the bioavailability of polyphenols remains an issue to be addressed, as it is known that, in most cases, only low concentrations can reach the site of activity, thus reducing their potential effects [26,27]. On the other hand, after ingestion, polyphenols are differentially absorbed in the gastrointestinal tract and can interact with the gut microbiota, modulating metabolites produced by bacterial fermentation [28].

Red oranges and olive leaf wastes were utilized as starting raw materials to produce standardized extracts referred to red orange extract (ROE) and olive leaf extract (OLE), respectively. The extracts were tested alone and in combination and displayed antioxidant activities in a concentration-dependent manner, as demonstrated with the FRAP assay, in agreement with other studies reporting free radical scavenging and strong antioxidant activity of waste-derived extracts [11,12].

A strong antioxidant capacity is usually associated with a reduction in lipid adipocyte storage since ROS generation mediates the adipogenesis process in vitro [29]. As observed in our experimental conditions, combination treatment exhibited a synergistic effect in reducing lipid droplet accumulation, confirming the extracts’ ability to influence adipogenesis and suggesting their potential anti-obesity effect.

The differentiation of pre-adipose cells in adipocytes necessitates the active synthesis of collagen. Nevertheless, excessive deposition of the extracellular matrix in adipose tissue is characteristic of fibrosis, which can result in dysfunctional adipose tissue [30]. Herein, we reported a potent anti-fibrotic effect of ROE and OLE at different time points, reflected by a reduction in soluble collagen release, in accordance with evidence on plant-derived bioactive compounds’ capacity to influence adipose tissue fibrosis biomarkers [31,32]. 

Obese and T2DM individuals often display elevated plasma levels of FFAs, particularly palmitic acid, which is the most prevalent saturated fatty acid in the bloodstream.

Palmitic acid has been identified as a negative regulator of insulin signaling by inhibiting insulin action in isolated hepatocytes, myocytes, and adipocytes. Metabolic byproducts of palmitic acid influence the activation of toll-like receptors, endoplasmic reticulum stress, and an increase in ROS generation [7].

To better characterize adipocytes’ inflammatory response, we present evidence on effects of ROE and OLE under PA treatment on mature adipocytes. Although PA represents a prevalent fatty acid that is not esterified as well as oleic acid, its administration did not result in increased oil red quantification in our model. On the other hand, PA caused the development of an inflammatory state, which affects adipocyte functionality, leading to a reduction in triglyceride accumulation, as reflected by reduced DGAT mRNA levels, and impaired fatty acid cellular import due to diminished FATP1–4 mRNA [33]. Subsequently, to confirm the anti-inflammatory effects of the extracts, gene levels related to inflammation, such as IL-6, HO-1, and COL1A1, and lipid metabolism, such as FAS, DGAT-1, DGAT-2, SREBP-1C, FATP-1, and FATP-4, were measured by qRT-PCR in differentiated adipocytes exposed to PA, highlighting their ability to counteract PA-induced metabolic alterations.

Several naturally derived bioactive molecules have been studied for their potential to inhibit IL-6 activity, which is positively associated with body mass index (BMI) and negatively affects insulin signaling [34,35].

The observed reduction in IL-6 levels following ROE and OLE treatment was associated with the upregulation of the inducible antioxidant enzyme HO-1, which has been linked to a reduction in metabolic syndrome incidence [36,37].

Low levels of HO-1 are linked to disease progression, while the induction of HO-1 has been shown to inhibit the evolution of metabolic diseases [38,39,40]. Natural compounds possessing antioxidant properties have become valuable sources of HO-1 inducers. Phenolic compounds, which are found in numerous food and medicinal plants, have been studied extensively for their antioxidant activity and other pharmacological effects [41]. As evidenced by previous results from the Sircol soluble assay, the downregulation of the COL1A1 gene confirms the antifibrotic effect of the extracts. These data indicate that the treatments possess strong anti-inflammatory, antioxidative, and antifibrotic properties. 

The transcription factor SREBP1 and its target gene FAS, both implicated in the regulation of fatty acid, cholesterol, and triglyceride synthesis, were significantly reduced following the combination treatment, confirming the anti-obesity effects of ROE and OLE.

As reported in other studies, reducing the activity of adipogenic transcription factors could be a promising strategy to prevent the enlargement of adipose tissue in the progression of obesity [10,42,43,44].

Numerous studies have shown that inhibition of DGAT1 can yield multiple beneficial effects on metabolic diseases [45,46,47], as observed in our experimental conditions. 

FATP enzymes have a crucial function in fatty acid uptake and metabolism. Consequently, changes in FATP expression within metabolic tissues have been identified as significant contributors to diet-induced obesity, hepatic steatosis, insulin resistance, and metabolic syndrome in both mice and humans. FATP1 exhibits high expression levels in hormone-sensitive tissues, such as WAT, where insulin is required for FATP1 to facilitate fatty acid (FA) uptake. FATP4 facilitates FA uptake not just in insulin-sensitive tissues but also in insulin-insensitive tissues [48,49,50], as in the case of our experimental condition, in which the widely renowned model of PA-induced insulin resistance determines the reduction of FATP levels. Impaired lipid trafficking caused by PA was recovered by the OLE and COMBO treatments, which were able to restore both FATP1–4 levels compared to the PA-challenged group, suggesting their potential in maintaining basal adipocyte lipid import. Taken together, these data highlight the ability to increase the cellular import of fatty acids for the beta oxidation process.

Based on the observed correlation between natural products and ferroptosis modulation as a potential strategy for the management of several diseases [51,52,53,54], we aimed to preliminary investigate the potential beneficial effects of ROE/OLE against erastin-induced cell death. Interestingly, we observed a different sensitivity to erastin in pre-adipocytes and mature adipocytes. Indeed, pre-adipocytes were more susceptible to oxidative cell death, which was rescued by the ROE and OLE treatments, proving their cytoprotective effects.

Even though erastin did not cause a reduction in cell viability in mature adipocytes, it was responsible for altering lipid metabolism, as shown by an increase in fat accumulation. The current results extend these observations by showing, for the first time, that ROE and OLE were able to prevent the erastin-induced hypertrophic condition.

## 5. Conclusions

Natural compounds have garnered considerable attention for their potential anti-obesity effects, and they have been extensively assessed for their preventive and/or therapeutic properties against obesity. The purpose of the present study was to investigate the effect of standardized extracts derived from red oranges and olive leaf waste, which are abundant in bioactive molecules, including polyphenols. We further confirmed the antioxidant activity of the ROE and OLE, and we assessed the extracts’ capacity to reduce lipid accumulation and collagen accumulation during adipogenesis, suggesting their potential anti-obesity and anti-fibrotic effects.

Chronic inflammation associated with obesity may represent a potential correlation with a ferroptotic mechanism. Based on this, we have preliminary investigated the anti-inflammatory and cytoprotective effects of extracts in an in vitro model of erastin-induced cell death.

Natural-derived products are conventional resources for drug discovery, and some of them have been clinically used against human disease; moreover, they have the potential to modulate the expression levels of ferroptosis-related factors.

Therefore, exploiting bioactive molecules that can be found in extracts such as ROE and OLE that modulate ferroptosis may be harnessed to facilitate the development of promising therapeutic strategies.

## Data Availability

The original contributions presented in the study are included in the article, further inquiries can be directed to the corresponding author.

## References

[1] Kawai T., Autieri M.V., Scalia R. (2021). Adipose tissue inflammation and metabolic dysfunction in obesity. Am. J. Physiol. Cell Physiol..

[2] Sabaratnam R., Svenningsen P. (2021). Adipocyte-Endothelium Crosstalk in Obesity. Front. Endocrinol..

[3] Mancini A., Imperlini E., Nigro E., Montagnese C., Daniele A., Orrù S., Buono P. (2015). Biological and Nutritional Properties of Palm Oil and Palmitic Acid: Effects on Health. Molecules.

[4] Pratelli G., Carlisi D., D’Anneo A., Maggio A., Emanuele S., Palumbo Piccionello A., Giuliano M., De Blasio A., Calvaruso G., Lauricella M. (2022). Bio-Waste Products of Mangifera indica L. Reduce Adipogenesis and Exert Antioxidant Effects on 3T3-L1 Cells. Antioxidants.

[5] Ali A.T., Hochfeld W.E., Myburgh R., Pepper M.S. (2013). Adipocyte and adipogenesis. Eur. J. Cell Biol..

[6] Armani A., Mammi C., Marzolla V., Calanchini M., Antelmi A., Rosano G.M., Fabbri A., Caprio M. (2010). Cellular models for understanding adipogenesis, adipose dysfunction, and obesity. J. Cell. Biochem..

[7] Amine H., Benomar Y., Taouis M. (2021). Palmitic acid promotes resistin-induced insulin resistance and inflammation in SH-SY5Y human neuroblastoma. Sci. Rep..

[8] Pérez-Torres I., Castrejón-Téllez V., Soto M.E., Rubio-Ruiz M.E., Manzano-Pech L., Guarner-Lans V. (2021). Oxidative Stress, Plant Natural Antioxidants, and Obesity. Int. J. Mol. Sci..

[9] Karri S., Sharma S., Hatware K., Patil K. (2019). Natural anti-obesity agents and their therapeutic role in management of obesity: A future trend perspective. Biomed. Pharmacother..

[10] Jakab J., Miškić B., Mikšić Š., Juranić B., Ćosić V., Schwarz D., Včev A. (2021). Adipogenesis as a Potential Anti-Obesity Target: A Review of Pharmacological Treatment and Natural Products. Diabetes Metab. Syndr. Obes..

[11] Sorrenti V., Burò I., Consoli V., Vanella L. (2023). Recent Advances in Health Benefits of Bioactive Compounds from Food Wastes and By-Products: Biochemical Aspects. Int. J. Mol. Sci..

[12] Burò I., Consoli V., Castellano A., Vanella L., Sorrenti V. (2022). Beneficial Effects of Standardized Extracts from Wastes of Red Oranges and Olive Leaves. Antioxidants.

[13] Ma W., Jia L., Xiong Q., Du H. (2021). Iron Overload Protects from Obesity by Ferroptosis. Foods.

[14] Zhao X., Si L., Bian J., Pan C., Guo W., Qin P., Zhu W., Xia Y., Zhang Q., Wei K. (2022). Adipose tissue macrophage-derived exosomes induce ferroptosis via glutathione synthesis inhibition by targeting SLC7A11 in obesity-induced cardiac injury. Free Radic. Biol. Med..

[15] Zhou D., Lu P., Mo X., Yang B., Chen T., Yao Y., Xiong T., Yue L., Yang X. (2023). Ferroptosis and metabolic syndrome and complications: Association, mechanism, and translational applications. Front. Endocrinol..

[16] Zhao Y., Li Y., Zhang R., Wang F., Wang T., Jiao Y. (2020). The Role of Erastin in Ferroptosis and Its Prospects in Cancer Therapy. Onco Targets Ther..

[17] Ballistreri G., Amenta M., Fabroni S., Consoli V., Grosso S., Vanella L., Sorrenti V., Rapisarda P. (2021). Evaluation of lipid and cholesterol-lowering effect of bioflavonoids from bergamot extract. Nat. Prod. Res..

[18] Consoli G.M.L., Forte G., Maugeri L., Consoli V., Sorrenti V., Vanella L., Buscarino G., Agnello S., Camarda M., Granata G. (2023). Near-Infrared-Responsive Choline-Calix [4]arene-Gold Nanostructures for Potential Photothermal Cancer Treatment. ACS Appl. Nano Mater..

[19] Raffaele M., Carota G., Sferrazzo G., Licari M., Barbagallo I., Sorrenti V., Signorelli S.S., Vanella L. (2019). Inhibition of Heme Oxygenase Antioxidant Activity Exacerbates Hepatic Steatosis and Fibrosis. Antioxidants.

[20] Vanella L., Consoli V., Burò I., Gulisano M., Giglio M.S., Maugeri L., Petralia S., Castellano A., Sorrenti V. (2023). Standardized Extract from Wastes of Edible Flowers and Snail Mucus Ameliorate Ultraviolet B-Induced Damage in Keratinocytes. Int. J. Mol. Sci..

[21] Ruiz-Ojeda F.J., Rupérez A.I., Gomez-Llorente C., Gil A., Aguilera C.M. (2016). Cell Models and Their Application for Studying Adipogenic Differentiation in Relation to Obesity: A Review. Int. J. Mol. Sci..

[22] Kruczkowska W., Gałęziewska J., Kciuk M., Gielecińska A., Płuciennik E., Pasieka Z., Zhao L.Y., Yu Y.J., Kołat D., Kałuzińska-Kołat Ż. (2024). Senescent adipocytes and type 2 diabetes—Current knowledge and perspective concepts. Biomol. Concepts.

[23] Palacios-Marin I., Serra D., Jimenez-Chillarón J., Herrero L., Todorčević M. (2023). Adipose Tissue Dynamics: Cellular and Lipid Turnover in Health and Disease. Nutrients.

[24] Saad B. (2023). A Review of the Anti-Obesity Effects of Wild Edible Plants in the Mediterranean Diet and Their Active Compounds: From Traditional Uses to Action Mechanisms and Therapeutic Targets. Int. J. Mol. Sci..

[25] Jack B.U., Malherbe C.J., Mamushi M., Muller C.J.F., Joubert E., Louw J., Pheiffer C. (2019). Adipose tissue as a possible therapeutic target for polyphenols: A case for Cyclopia extracts as anti-obesity nutraceuticals. Biomed. Pharmacother..

[26] Rana A., Samtiya M., Dhewa T., Mishra V., Aluko R.E. (2022). Health benefits of polyphenols: A concise review. J. Food Biochem..

[27] D’Archivio M., Filesi C., Varì R., Scazzocchio B., Masella R. (2010). Bioavailability of the polyphenols: Status and controversies. Int. J. Mol. Sci..

[28] Zhang W., Qi S., Xue X., Al Naggar Y., Wu L., Wang K. (2021). Understanding the Gastrointestinal Protective Effects of Polyphenols using Foodomics-Based Approaches. Front. Immunol..

[29] Castro J.P., Grune T., Speckmann B. (2016). The two faces of reactive oxygen species (ROS) in adipocyte function and dysfunction. Biol. Chem..

[30] Gregoire F.M., Smas C.M., Sul H.S. (1998). Understanding adipocyte differentiation. Physiol. Rev..

[31] Taheri A., Mobaser S.E., Golpour P., Nourbakhsh M., Tavakoli-Yaraki M., Yarahmadi S. (2023). Hesperetin attenuates the expression of markers of adipose tissue fibrosis in pre-adipocytes. BMC Complement. Med. Ther..

[32] Li X., Li J., Wang L., Li A., Qiu Z., Qi L.W., Kou J., Liu K., Liu B., Huang F. (2016). The role of metformin and resveratrol in the prevention of hypoxia-inducible factor 1α accumulation and fibrosis in hypoxic adipose tissue. Br. J. Pharmacol..

[33] Leamy A.K., Hasenour C.M., Egnatchik R.A., Trenary I.A., Yao C.H., Patti G.J., Shiota M., Young J.D. (2016). Knockdown of triglyceride synthesis does not enhance palmitate lipotoxicity or prevent oleate-mediated rescue in rat hepatocytes. Biochim. Biophys. Acta.

[34] Harmalkar D.S., Sivaraman A., Nada H., Lee J., Kang H., Choi Y., Lee K. (2024). Natural products as IL-6 inhibitors for inflammatory diseases: Synthetic and SAR perspective. Med. Res. Rev..

[35] Khodabandehloo H., Gorgani-Firuzjaee S., Panahi G., Meshkani R. (2016). Molecular and cellular mechanisms linking inflammation to insulin resistance and β-cell dysfunction. Transl. Res..

[36] Liu C., Xu X., He X., Ren J., Chi M., Deng G., Li G., Nasser M.I. (2023). Activation of the Nrf-2/HO-1 signalling axis can alleviate metabolic syndrome in cardiovascular disease. Ann. Med..

[37] Son Y., Lee J.H., Chung H.T., Pae H.O. (2013). Therapeutic roles of heme oxygenase-1 in metabolic diseases: Curcumin and resveratrol analogues as possible inducers of heme oxygenase-1. Oxid. Med. Cell Longev..

[38] McClung J.A., Levy L., Garcia V., Stec D.E., Peterson S.J., Abraham N.G. (2022). Heme-oxygenase and lipid mediators in obesity and associated cardiometabolic diseases: Therapeutic implications. Pharmacol. Ther..

[39] Drummond G.S., Baum J., Greenberg M., Lewis D., Abraham N.G. (2019). HO-1 overexpression and underexpression: Clinical implications. Arch. Biochem. Biophys..

[40] Consoli V., Sorrenti V., Grosso S., Vanella L. (2021). Heme Oxygenase-1 Signaling and Redox Homeostasis in Physiopathological Conditions. Biomolecules.

[41] Hahn D., Shin S.H., Bae J.S. (2020). Natural Antioxidant and Anti-Inflammatory Compounds in Foodstuff or Medicinal Herbs Inducing Heme Oxygenase-1 Expression. Antioxidants.

[42] Moseti D., Regassa A., Kim W.K. (2016). Molecular Regulation of Adipogenesis and Potential Anti-Adipogenic Bioactive Molecules. Int. J. Mol. Sci..

[43] Xiao X., Song B.L. (2013). SREBP: A novel therapeutic target. Acta Biochim. Biophys. Sin..

[44] Tang J.J., Li J.G., Qi W., Qiu W.W., Li P.S., Li B.L., Song B.L. (2011). Inhibition of SREBP by a small molecule, betulin, improves hyperlipidemia and insulin resistance and reduces atherosclerotic plaques. Cell Metab..

[45] Huang J.S., Guo B.B., Wang G.H., Zeng L.M., Hu Y.H., Wang T., Wang H.Y. (2021). DGAT1 inhibitors protect pancreatic β-cells from palmitic acid-induced apoptosis. Acta Pharmacol. Sin..

[46] Zhu G., Luo Y., Xu X., Zhang H., Zhu M. (2019). Anti-diabetic compounds from the seeds of Psoralea corylifolia. Fitoterapia.

[47] Imran M., Arshad M.S., Butt M.S., Kwon J.H., Arshad M.U., Sultan M.T. (2017). Mangiferin: A natural miracle bioactive compound against lifestyle related disorders. Lipids Health Dis..

[48] Lobo S., Wiczer B.M., Smith A.J., Hall A.M., Bernlohr D.A. (2007). Fatty acid metabolism in adipocytes: Functional analysis of fatty acid transport proteins 1 and 4. J. Lipid Res..

[49] Li H., Herrmann T., Seeßle J., Liebisch G., Merle U., Stremmel W., Chamulitrat W. (2022). Role of fatty acid transport protein 4 in metabolic tissues: Insights into obesity and fatty liver disease. Biosci. Rep..

[50] Du J., Zhu Y., Yang X., Geng X., Xu Y., Zhang M. (2024). Berberine attenuates obesity-induced insulin resistance by inhibiting miR-27a secretion. Diabet. Med..

[51] Deng L., Tian W., Luo L. (2024). Application of natural products in regulating ferroptosis in human diseases. Phytomedicine.

[52] Zuo H.L., Huang H.Y., Lin Y.C., Liu K.M., Lin T.S., Wang Y.B., Huang H.D. (2023). Effects of Natural Products on Enzymes Involved in Ferroptosis: Regulation and Implications. Molecules.

[53] El Hajj S., Canabady-Rochelle L., Gaucher C. (2023). Nature-Inspired Bioactive Compounds: A Promising Approach for Ferroptosis-Linked Human Diseases?. Molecules.

[54] Zheng K., Dong Y., Yang R., Liang Y., Wu H., He Z. (2021). Regulation of ferroptosis by bioactive phytochemicals: Implications for medical nutritional therapy. Pharmacol. Res..

